# Carbapenemase screening in an Irish tertiary referral hospital: Best practice, or can we do better?

**DOI:** 10.1016/j.infpip.2020.100100

**Published:** 2020-11-18

**Authors:** S. Fahy, J.A. O'Connor, D. O'Brien, L. Fitzpatrick, M. O'Connor, J. Crowley, M. Bernard, R.D. Sleator, B. Lucey

**Affiliations:** a Department of Clinical Microbiology, Mercy University Hospital, Cork, Ireland; b Department of Biological Sciences, Cork Institute of Technology, Bishopstown, Cork, Ireland; c Infection Prevention & Control Department, Mercy University Hospital, Cork, Ireland

**Keywords:** Carbapenemase producing enterobacterales, Surveillance, MDRO screening, Epidemiology, Algorithm, CPE screening

## Abstract

**Background:**

Carbapenems are a family of end line antibiotics with increasing levels of resistance that are a cause for concern.

**Aim:**

To ascertain whether the CPE screening programme employed in an acute tertiary hospital is fit for purpose.

**Method:**

We outlined the current working algorithm employed using a universal screening programme over a 26-month screening period. Rectal swabs are cultured on arrival. Those with suspicious growth are further investigated using NG-Carba 5 lateral flow tests and Vitek 2.0 sensitivity cards. These practices were compared with NHS guidelines.

**Findings & Conclusions:**

In all, 53 true positives were detected from 45 patients since the screening was implemented in early 2018 (46 OXA-48, 6 KPC, 1 NDM). As the rate of screening increased, the number of positive screens decreased over time. There were a lot of similarities between the HSE guidelines and the published NHS CPE toolkit. It was evident that there is no standard practice being employed across all hospitals. Comparing the MUH to national guidelines it appears to be quicker and more effective with universal screening in place at reducing the potential contacts and identifying carriers. Cost analysis indicates that the need to confirm all positive strains in a reference lab is costly, unnecessary and time consuming. There are adequate confirmatory tests available in-house for routine positive screens. It was concluded that infection prevention and control are key to identifying and controlling possible outbreaks in a hospital setting.

## Introduction

The evolution of antibiotic resistance is having an extensive and significant effect on society [1]. Resistance rates continue to rise, while antibiotic discovery has decreased substantially [2]. Carbapenems are a family of last line antibiotics that are administered intravenously to treat serious infections [3]. Resistance to these agents is increasing through the production of Carbapenemases, which hydrolyse the penicillin ring, rendering the antibiotic ineffective [4]. Carbapenemase transmission has been recorded over the past two decades, with global dissemination becoming a cause for significant public concern [5]. Invasive Carbapenemase producing *Enterobacterales* infections were made notifiable in Ireland in 2011, since then, there has been a steady increase in the incidence of CPE in Ireland, with an overall increase of 26% in 2018 (449–565 cases) [6]. CPE are most commonly spread faecal-orally [7] and with improper hand hygiene and poor environmental cleaning can be silently transferred from healthcare workers to patients within healthcare facilities, particularly if an effective screening programme is not in place. At present, it is advised that all presumptive new CPE isolates detected in local hospitals in Ireland be confirmed by the reference laboratory located in the Microbiology Department of Galway University Hospital (GUH) [8]. The most prominent CPE genes detected in invasive bloodstream infections in Ireland between 2012 and 2018 were OXA-48, KPC, NDM, VIM, IMP [9].

Initially, United States Centre of Disease Control (CDC) recommended culture-based CPE screens; using broth enrichment in tryptone soy broth (TSB), followed by culture onto MacConkey agar with a carbapenem disc. However, this protocol is both time consuming (approx. 48hrs) and labour intensive, making it impractical for routine screening, particularly in a busy hospital laboratory [10]. Some improvements have been observed using commercially available culture plates, particularly in terms of decreased turnaround times (approx. 24hrs) and increased sensitivity/specificity for CPE producers; mSuperCarba (CHROMagar) media, for example has shown an increased sensitivity rate of 83% compared to 69% for MacConkey culture with an imipenem disc [11]. Lateral flow testing has also been utilised as a confirmatory test in several European clinical microbiology laboratories [12,13]. A recent immunochromatographic development is the NG-Test CARBA 5 (NG Biotech, Guipry, France) which detects the five most prominent Carbapenemases (OXA-48, KPC, NDM, VIM, IMP) in 15 minutes from cultured colonies, at room temperature [14]. A recent study by Keiffer *et al.*, [15] reported a 100% detection specificity/sensitivity of known Carbapenemase producers in a Swedish reference laboratory. Thus, accurate and timely antibiotic resistant patterns, obtained using either manual or automatic methods, are essential when screening for CPEs. Prompt identification of CPE is essential to implement transmission-based infection prevention and control precautions and limit the potential spread to other patients.

The Mercy University Hospital (MUH) is a 271-bed tertiary referral centre in Cork, Ireland, which undertakes Carbapenemase screening for all patients being admitted for an overnight admission, and weekly thereafter. A national patient electronic system is still not in place in Ireland. Information regarding the patient's history of MDRO colonisation is available to view locally using an electronic patient record system (iPMS) in the South/South West hospital group. This information is managed by infection prevention and control teams in each hospital.

The screening protocol was implemented as part of a co-ordinated outbreak response early in 2018. Rectal swabs are the preferred screening specimen, with faecal samples accepted if swabs are not possible.

To date, there have been in excess of 14,000 screens carried out in the MUH, from which 53 true positives were detected from 45 patients. Thirty five of these patients were inpatients at time of sampling. This study describes the emergence of CPE, providing a point prevalence study among MUH inpatients. We also describe a comprehensive CPE screening programme and evaluate the current working algorithm in (MUH), in comparison to both the recent Health Protection Surveillance Centre (HSPC) Guidelines and the United Kingdom Health Service (NHS) standard [16].

The first MUH acquired CPE case was identified in December 2017 with a further four cases identified in January 2018. Following initial targeted CPE surveillance screening a decision was made to implement a policy of universal CPE screening in January 2018. This demonstrated a low CPE prevalence of 0.28% among those screened. All in-house positive CPE isolates and negative CPE isolates with unexpected reduced susceptibilities to carbapenems (i.e. those that do not exhibit any inherent resistance to carbapenems) were confirmed by the national reference laboratory.

## Materials and methods

The study was conducted in the Clinical Microbiology laboratory in the Mercy University Hospital (MUH). This paper examines the results for the MUH CPE screening programme over a 26-month period, and the MUH CPE protocol is compared to national and international best practice guidelines [8,17]. Ethical approval was granted by the Clinical Research Ethics Committee, Cork. (Review reference number ECM 4 (q))

**Figure 1:**
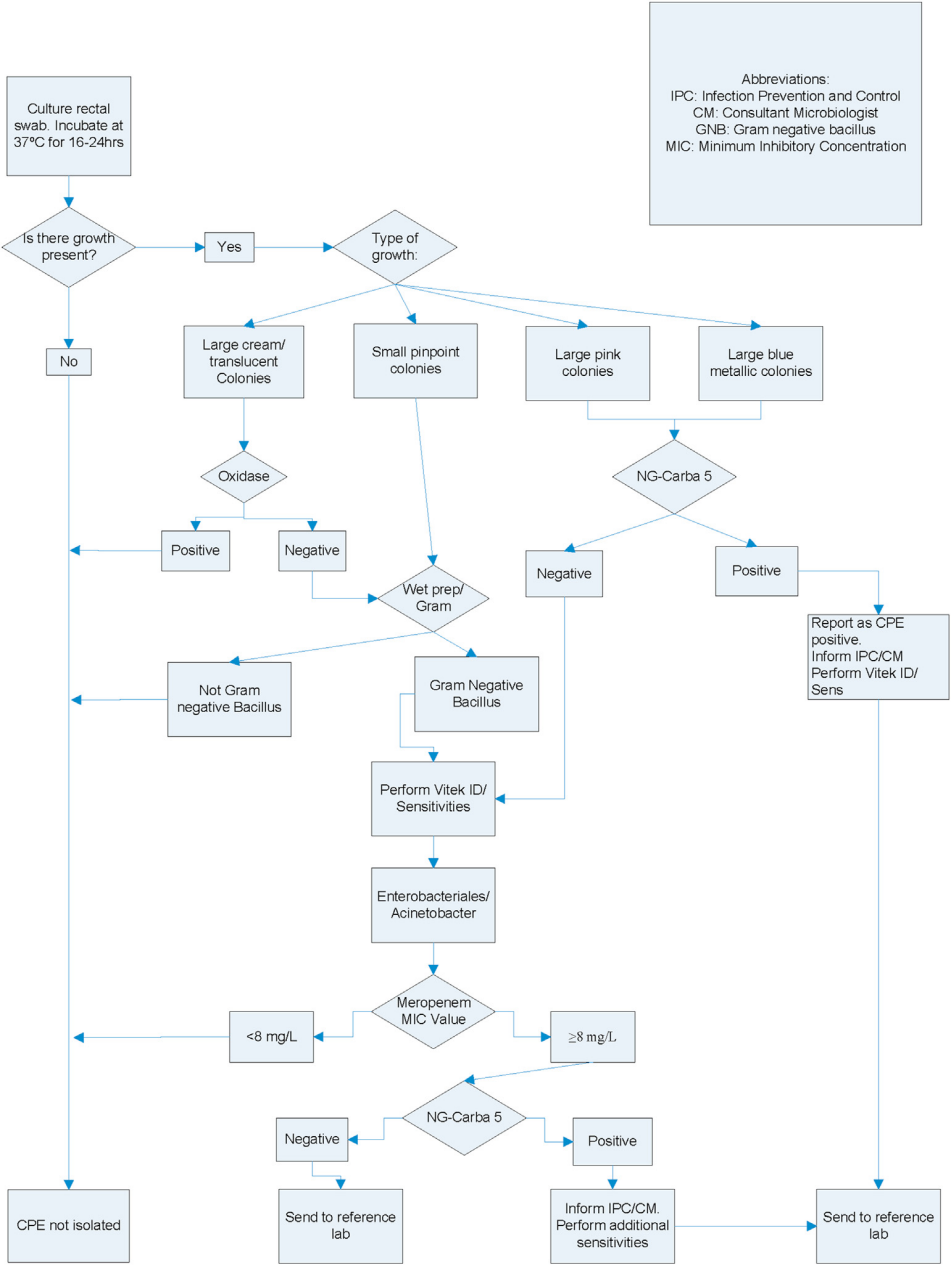
CPE working algorithm, Mercy University Hospital Microbiology Department.

The current algorithm is shown in Figure 1. Briefly, the rectal swab is cultured onto an mSuperCarba chromogenic CRE agar and incubated for a period of 16–24 hours, in accordance with manufacturer's guidelines. The plates are examined post incubation and those with visible colony growth are subject to further investigation, as outlined in Figure 1. Those with no growth are reported as “CPE not isolated”. Colonies are tested for oxidase activity to rule out *Pseudomonas* spp. It should be noted that those colonies that are oxidase-negative are Gram stained to confirm that the suspect isolate is a Gram-negative bacillus*.* Gram-negative colonies are then tested for presence of CPE determinants using the NG-Test CARBA 5 immunochromatographic assay. Positive NG-Test CARBA 5 results are reported as CPE isolated and immediately reported to the Consultant Microbiologist/Infection Prevention and Control Nurses to allow for patient isolation and to reduce the number of potential contacts. Positive isolates are sub-cultured in duplicate onto a nutrient slope; one to be stored at -70°C for any further investigations, the second to be referred to the National Reference Laboratory, located in the Microbiology Department of Galway University Hospital (GUH). This reference service is offered to all clinical laboratories in Ireland. The user guide is available to all laboratories to aid in the detection of minor and major CPE outbreaks [8].

Data gathered for this study was collected using the laboratory information system APEX. Positive isolates are kept on a queue for authorisation. Patient's electronic notepads are also flagged once a CPE has been confirmed for infection control purposes.

## Results

There were 53 confirmed CPE cases in MUH in the period of study. The organism identifications, meropenem Minimum Inhibitory Concentration and in-house details are recorded in Table I. Identification was conducted using Vitek 2.0 with meropenem MICs being carried out manually as required. Less than 1% of OXA-48 isolates in this study are resistant to meropenem with all 6 *Klebsiella pneumoniae* Carbapenemase (KPC) strains and a single New-Delhi metallo-β-lactamase (NDM) showing resistant patterns of >8mg/L. All in-house positives correlated with reference laboratory findings.

**Table I:** Description of positive Carbapenemase producing (CPE) isolates, reported from the Microbiology Department of the Mercy University Hospital since 2017

Organism identification (no.)	In-house NG-Test CARBA 5 result (no.)	Meropenem interpretation (EUCAST S/I/R) result (no.)	Reference laboratory result (no.)
*Escherichia coli* (31)	NDM (1), OXA-48 (30)	NDM: R (1), OXA-48: S (30)	NDM (1), OXA-48 (30)
*Klebsiella pneumoniae* (15)	KPC (6), OXA-48 (9)	KPC: R (6), OXA-48: I (1), OXA-48: S (8)	KPC (6), OXA-48 (9)
*Enterobacter cloacae* (3)	OXA-48 (3)	OXA-48 S (2), OXA-48 I (1)	OXA-48 (3)
*Klebsiella oxytoca* (2)	OXA-48 (2)	OXA-48 S (1), OXA-48 R (1)	OXA-48 (2)
*Citrobacter freundii* (2)	OXA-48 (2)	OXA-48 S (2)	OXA-48 (2)

S= Sensitive, I= Intermediate, R= Resistant, NDM= New-Delhi metallo-β-lactamase, KPC= *Klebsiella pneumoniae* Carbapenemase, VIM= Verona integron-mediated metallo-β-lactamase, IMP= active-on-imipenem, OXA-48= oxacillinase

**Figure 2:**
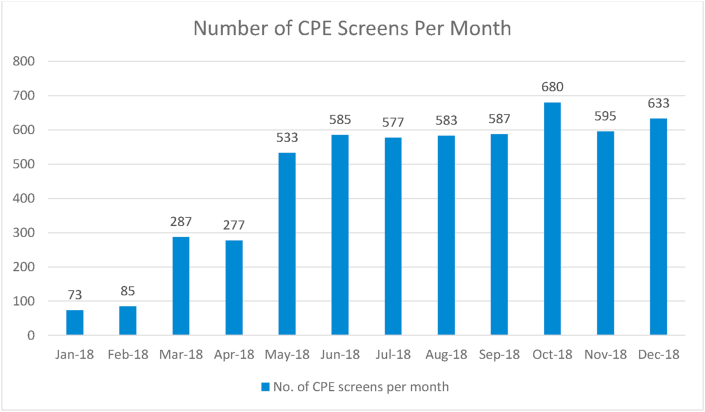
Number of CPE screens carried out per month in MUH. (Infection Prevention & Control Department, MUH).

**Figure 3:**
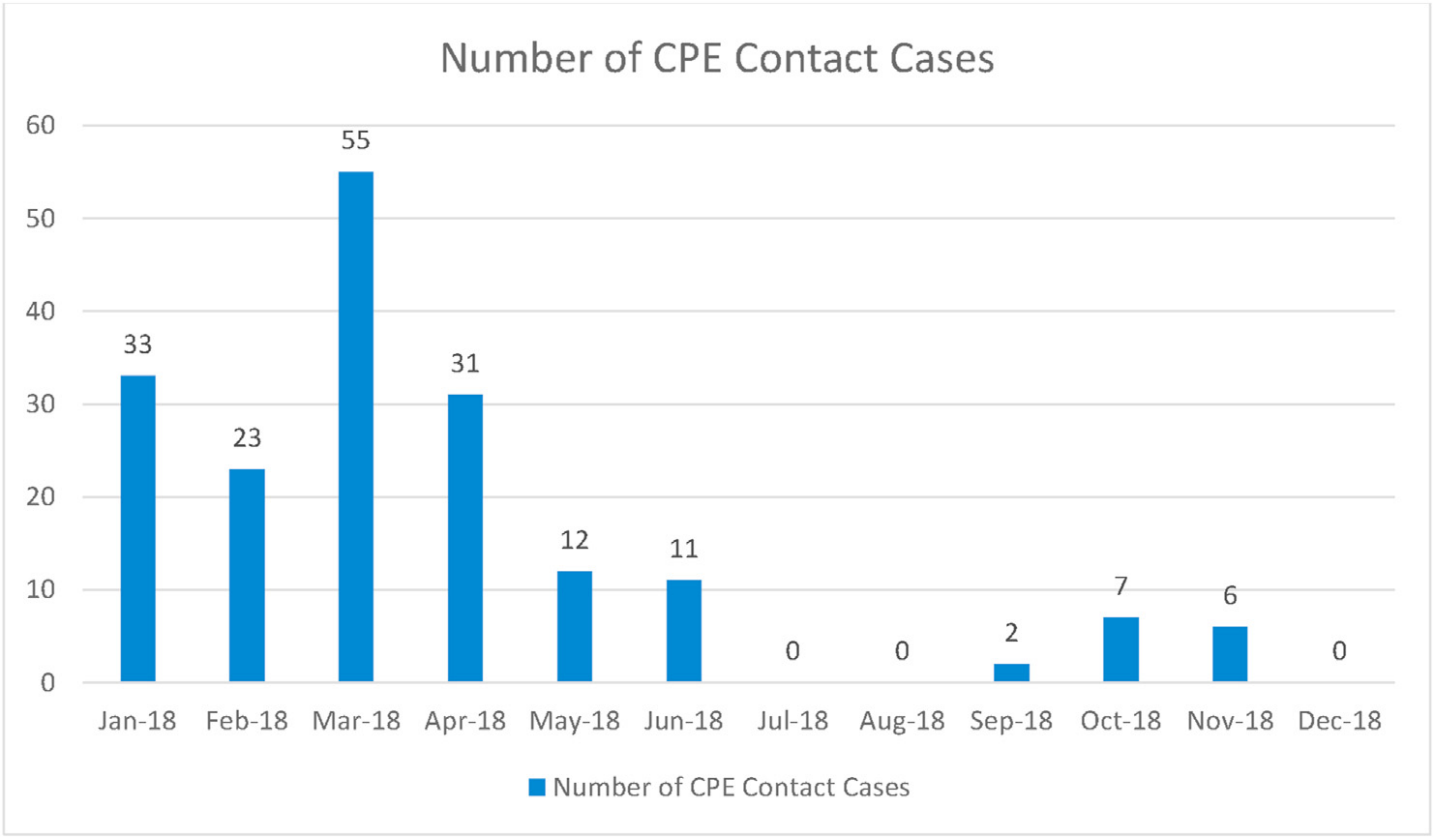
Number of New CPE Contacts MUH 2018 (Infection Prevention & Control Department, MUH).

The MUH progressed from high risk screening only and employed a universal CPE screening programme on all in-patients since April 2018. Although this was not an official recommendation, it was decided by hospital management to implement it on a trial basis, the results of which are shown in Figure 2, Figure 3 below.

## Discussion

Should CPE transmission not be controlled appropriately, it will have substantial health and financial consequences on hospital settings [18]. The UK's National Health Service (NHS) published a CPE toolkit in December 2013 [16] as a guideline for NHS hospitals to establish protocols to control the spread of CPE. As there are inter-laboratory method variations in the UK as outlined by Berry *et al.* [19], guidelines recommend methods used should have ‘*demonstrated performance at least equivalent to plating on to a commercially prepared agar specifically recommended for this purpose*’ [20]. Surveys were distributed to several NHS trust hospitals to gather data on different screening algorithms and CPE rates; 34/153 (22.2%) labs returned both surveys with a wide variety of methods in place. Phenotypic tests accounted for 55.6% of screening. 12/153 (7.8%) labs were using molecular testing to screen. A total of 94.4% hospitals reported the most common reason for screening being a recent history of travel. Comparative analysis based on both published CPE toolkits by the Health Service Executive (HSE) and NHS respectively has highlighted some differences in standard screening practices. HSE-governed public hospitals are required to target screen certain patient cohorts e.g immunocompromised/at risk patients [21]. The NHS toolkit states that if the patient has no known risk, no screening is conducted [16]. In some contrast to these guidelines in the MUH, wholesale screening is part of the admission protocol since April 2018. This reduces the need to risk assess patients on admission. The HSE toolkit has been updated annually with the NHS toolkit now due for its first revision since being published in 2013. Although there are some differences noted between NHS and HSE guidelines, there are also many similarities in screening and management protocols proposed across both systems. The regions reporting the highest number of CPE isolated were also carrying out the most screens per hospital, which may suggest the use of less effective screening protocols by others or no screening at all. NHS reports that patients with 3 negative screens during a stay are not re-screened, contrasting with MUH practice whereby screening is conducted weekly for the duration of stay. Our findings show that of the 53 true positives detected in MUH, four of these derived from weekly screens where the patient had previously tested negative during the same hospital admission. These results ranged from one to two previous negative screens. Depending on the length of a patient stay, we suggest that weekly screening is merited; as it ensures that CPE positives will be detected once weekly, at least, on all in-patients, thus potentially reducing the chance of transmission.

A total of 76% of NHS hospitals reported chromogenic agar being used as their first step, which is in-line with MUH practice and 42% of labs reported confirming positive detections in-house by molecular (24%) or phenotypic (56%) testing without isolates being sent to a reference centre. MUH refers all positive screens for confirmatory testing to Galway University Hospital (GUH) despite the availability of local confirmatory methods including phenotypic and, occasionally, molecular confirmation. We suggest that local confirmation should be made of putative positive test results.

As outlined in Figure 1, all culture-indicated positives are investigated according to protocol agreed in MUH. Gram-negative bacilli are tested using the NG-Test CARBA 5 lateral flow quick kit. All 53 confirmed positives have been successfully identified using this method. All culture positive, lateral flow negative isolates are worked up in accordance to Figure 1. Vitek susceptibilities are analysed to see what action is required. The reason for the breakthrough growth in some cases is unknown and to date all isolates with reduced susceptibility to meropenem with a negative lateral flow results have been confirmed as not harbouring any of the commonly encountered CPE genes by the reference laboratory. A recent change which was prompted by Galway University Hospital, to the CPE algorithm, resulted in the meropenem MIC criterion increasing from ≥0.25mg/L to ≥8mg/L for isolate referral [8]. This will see a reduction in the workload and cost for the laboratory. As can be noted from Table I above, 3/53 (5.7%) isolates had a meropenem ≥8mg/L, however all isolates with a meropenem value <8mg/L had been correctly detected using the NG-Test CARBA 5 lateral flow testing. There is not yet enough clinical evidence as to why these breakthrough strains (culture positive, lateral flow negative) are growing on the (in-date) chromogenic agar. Some, though not all, are producing high level AmpCs or ESBLs which have been shown to grow on commercial CPE media [22]. MUH is confident in detecting all prominent CPE isolates including OXA-48, VIM, IMP, NDM, KPC. However, lesser known CPE genes may go undetected if the meropenem value happens to be <8mg/L. A recent study noted when using mSupercarba media, that a decreased specificity for the identification of KPC and OXA-48 occurred due to over expressed AmpC genes and porin loss [23]. This is certainly an area that needs further investigation to reduce time and labour costs in the future.

The MUH universal screening policy ensured a quicker and more effective response to potential outbreaks. As screening rates increased, i.e. all-over-night stays excluding Emergency Department (ED), the contact numbers of positive patients decreased significantly. As can be seen in Figure 2, Figure 3 above, at a screening rate of 89% of admissions there were 0 reported cases of CPE in December 2018. This suggests the MUH policy of universal screening has had a positive impact in reducing the amount of potential contacts from asymptomatic carriers.

When comparing the MUH response to CPE on a global scale it would mirror the results Israel produced combating the fight against antimicrobial resistance genes. Israel implemented a national CPE screening policy in 2007 [24] which increased screening and reporting/tracing of contacts guided by a national plan to overcome a widespread outbreak of CPE. They have, to-date, been the only middle-eastern Country to prove successful in controlling the spread of CPE in hospitals and care facilities [25]. With the fast moving and large number of refugees fleeing Middle Eastern Countries it is important that CPE isolates are not easily transferred to other countries unknowingly.

Although it is difficult to estimate the cost of a Carbapenemase outbreak to each hospital, costs that are commonly encountered with CPE includes extended length of stay, staff time, drug and diagnostic costs [26]. The total cost of a routine laboratory investigation (reagents only) for positive CPE isolates is approximately €30, whilst in-house molecular testing may also cost €50 per test. It is not feasible to test samples in a low prevalence setting, daily using molecular methods owing to expense, which leads to the use of more fiscally responsible options, such as lateral flow kits, manual testing with meropenem and antibiotic susceptibility cards. However, molecular testing is a viable option where the result is required urgently, when a patient is at a high risk of potentially carrying CPE while being in contact with other patients in multiple occupancy wards. Some cartridge based molecular technologies, for example GeneXpert Carba-R (Cepheid) require hands-on time of less than a minute and this latter option offers results in approximately 48 minutes [27]. This suggests that if positives could be confirmed using the Gene Xpert Carba-R this would serve as a second confirmation test and samples would not have to be sent to the reference laboratory. In a recent study the GeneXpert Carba R test showed a 100% sensitivity rate and 98.1% specificity when used to confirm carbapenem producing Gram-negatives [28]*.* With the addition of courier costs and repeat testing (including organism identification) by the reference laboratory which costs a minimum of €50, it is our recommendation that there is enough confidence in in-house testing to confirm CPE isolates locally, this reduces the labour, financial and time cost which thereby, reduces the overall cost on a national level by >€5,000 on the 53 true positives and negatives with meropenem MICs >8 (mg/L) detected in the current study. A study conducted calculated the effectiveness of screening in the United States. It was recorded that at a prevalence of >0.3%, screening was cost effective to hospitals compared to cost incurred when colonisers go undetected [29]. A data bottleneck of whole genome sequence analysis is already a global problem [30], which again highlights the work being carried out unnecessarily by reference laboratories as a result of inappropriate referrals.

## Conclusion

The Microbiology Department in the MUH appear to be over-working putative CPE samples; suggesting that there is space to reduce the work required whilst still having confidence in catching all true CPE positives. In the current study we report 100% concordance between the results attained in-house and the reference laboratory (for both confirmed CPE positives and negatives). While this increases our confidence in the results attained in-house the findings suggest that it is not necessary to send all “CPE negative” isolates to the reference laboratory; indicating that a change in algorithm would significantly reduce complexity while providing the same results. We have demonstrated the impact of a universal CPE screening programme as a method of ending uncontrolled transmission and a valuable tool in preventing its re-emergence. As can be seen with COVID-19, a worldwide pandemic, infection prevention and control departments are an invaluable resource in all hospitals to implement procedures that ensure widespread infection is not common practice. The key to control is detecting asymptomatic carriers when it comes to CPE and many other hospital wide infections.

## Credit author statement

S. Fahy: Primary author and researcher.

J. A. O'Connor: Study, design, supervision, manuscript preparation.

D. O'Brien: Study, design, supervision, manuscript preparation.

L. Fitzpatrick: Provision of data, study, design.

M. O'Connor: Provision of data.

J. Crowley: Provision of data.

M. Bernard: Provision of data.

R. D. Sleator: Corresponding author, primary supervisor, study, design, supervision, manuscript preparation.

B. Lucey: Primary supervisor, study, design, supervision, manuscript preparation.

## Abbreviations

CPE: Carbapenemase producing Enterobacterales
GUH: Galway University Hospital
CDC: Centre of Disease Control
TSB: Tryptone soy broth
MUH: Mercy University Hospital
HSPC: Health Protection Surveillance Centre
NHS: National Health Service
IPC: Infection Prevention and Control
CM: Consultant Microbiologist
GNB: Gram negative bacillus
MIC: Minimum Inhibitory Concentration
NDM: New Delhi metallo-β-lactamase
KPC: *Klebsiella pneumoniae* Carbapenemase
VIM: Verona integron-mediated metallo-β-lactamase
IMP: active-on-imipenem
OXA-48: Oxacillinase
HSE: Health Service Executive
MEM: meropenem
ED: Emergency Department
